# Beyond Open Big Data: Addressing Unreliable Research

**DOI:** 10.2196/jmir.3871

**Published:** 2014-11-11

**Authors:** Edward T Moseley, Douglas J Hsu, David J Stone, Leo Anthony Celi

**Affiliations:** ^1^Division of Vaccine ResearchBeth Israel Deaconess Medical CenterBoston, MAUnited States; ^2^Division of Pulmonary, Critical Care and Sleep MedicineDepartment of MedicineBeth Israel Deaconess Medical CenterBoston, MAUnited States; ^3^UVA Center for Wireless HealthDepartments of Anesthesiology and Neurological SurgeryUniversity of VirginiaCharlottesville, VAUnited States; ^4^Center for Wireless HealthUniversity of VirginiaCharlottesville, VAUnited States; ^5^Institute for Medical Engineering & ScienceMassachusetts Institute of TechnologyCambridge, MAUnited States

**Keywords:** open data, unreliable research, collaborative learning, knowledge discovery, peer review, research culture

## Abstract

The National Institute of Health invests US $30.9 billion annually in medical research. However, the subsequent impact of this research output on society and the economy is amplified dramatically as a result of the actual medical treatments, biomedical innovations, and various commercial enterprises that emanate from and depend on these findings. It is therefore a great concern to discover that much of published research is unreliable. We propose extending the open data concept to the culture of the scientific research community. By dialing down unproductive features of secrecy and competition, while ramping up cooperation and transparency, we make a case that what is published would then be less susceptible to the sometimes corrupting and confounding pressures to be first or journalistically attractive, which can compromise the more fundamental need to be robustly correct.

## Under the Magnifying Lens: The Reliability of Medical Research

It ought to be remembered that there is nothing more difficult to take in hand, more perilous to conduct, or more uncertain in its success, than to take the lead in the introduction of a new order of things. Because the innovator has for enemies all those who have done well under the old conditions, and lukewarm defenders in those who may do well under the new.

Niccolo Machiavelli

The reliability of research is coming under increasing scrutiny. Over the past several years, editorials and entire issues of journals, including *British Medical Journal, The Lancet,* and even *The Economist* have highlighted the discordance between the amount of money invested in the biomedical research enterprise with the lack of reliability in published studies [1-4]. Despite the peer-review process, there is a fundamental problem with the reliability of a disturbing amount of scientific, particularly biomedical, research. The problem is basically two-fold and lies in the unreliability of what does get published and the inability of other researchers to know what is not getting published. False positive research results are successfully published with alarming frequency. Replication is intrinsically difficult for a variety of reasons including limited access to original data, intellectual property issues, the general perception that replication is not doing original (and rewarding) work, and also the possible appearance of being defiant to more senior authority. Of course, work that is never published cannot be examined or replicated. When re-examination does occur, irreproducibility is surprisingly rampant. Furthermore, the peer-review system is less robust than often assumed, and there are enormous career structure-related pressures to publish. It is remarkably easy for well-meaning but perhaps imperfectly objective researchers to be duped by false positive results that are generated by technical laboratory execution issues (including simple inconsistency) and faulty statistical analyses. The latter lies in inadequately powered studies, unlikely hypotheses being tested, lack of proper blinding, and the bias towards reporting and publishing something new. Wolfgang Pauli, the eminent physicist, might have classified this kind of work under his most brutal characterization of sloppy thinking, “It is not only not right, it is not even wrong,” or even better in the original German, “Das ist nicht nur nicht richtig, es ist nicht einmal falsch!” [5].

In the current research reward system, sensational positive findings—some, perhaps even the majority, of dubious reliability—are overvalued, while potentially important negative findings are underreported. In the words of a University of Virginia psychologist Brian Nosek, “There is no cost to getting things wrong. The cost is not getting them published” [6]. This underreporting may be due to several reasons including the reluctance of journals to publish negative results because they are perceived as intrinsically less interesting and important; the lower importance given to negative results in the sphere of promotions, awards, and grants; and unconscious or conscious self-censorship when negative results contradict a personal or industrial research agenda.

High-quality systematic reviews and meta-analyses are considered the strongest form of medical evidence. By including only published studies with sound methodology and robust analysis, the noise from irreproducible or improperly conducted research is diminished. However, systematic reviews do not adequately address publication bias. As exemplified by the drawn out Cochrane review of the neuraminidase inhibitors [7], this approach to evidence creation will be reliable only if there is access to all clinical trial data, and not only the ones that were analyzed and presented in publications. After finally getting access to previously unreleased studies (20 from Roche and 24 from GlaxoSmithKline), Cochrane Library found that the benefits of oseltamivir and zanamavir in the prevention and treatment of influenza had been overstated in previous meta-analyses [8].

More alarmingly, systematic reviews shed some light on the inefficiency of research in providing clinical guidelines. In a cross-sectional study of more than 1000 systematic reviews in the Cochrane Library across all 50 Collaborative Review Groups, 96% recommended further research to fully evaluate the intervention in question [9].

We have previously commented on the use of the vast amounts of data generated in the critical care setting to provide population-based data-driven care of individual patients [10]. Big data is an all-encompassing term for any collection of datasets so large and complex that it becomes difficult to process using traditional data processing applications. It has become an increasingly important element of research in many scientific areas such as astronomy [11], chemistry [12], microbiology [13], molecular biology [14], and physics [15]. Given the availability and use of these data and the dependency of a variety of enterprises, including clinical practice, on data analytics, there is justifiable concern regarding the issue. In addition, the use of increasingly big(ger) data will only further augment the noise resulting from these biases and problems that currently plague the scientific literature. We share these concerns and propose measures to mitigate the risk and improve the reliability and efficiency of the research enterprise.

## From Open Data to Collaborative Learning

We suggest building on a system, previously described to some extent, where data and methods are freely shared among different groups of investigators addressing the same or similar questions [16,17]. Clearly, the infrastructure for this sharing process would rest extensively on carefully engineered Internet-based processes. The Internet has become such a quietly ubiquitous factor in our lives that we may forget to explicitly acknowledge the huge fundamental impact that it has had and is most likely to continue having on information storage and exchange, and in the process dramatically expanding our problem-solving ability and increasing our combined brainpower [18]. Such processes would be created and implemented to validate and build on each group’s findings. Complete data interoperability would be required; this has been a particular issue with clinical data that emanate from the silos of different vendors. An open culture of sharing data would require a paradigm shift whereby individual research groups no longer compete for publication and funding. Research would be conducted with laboratories cooperatively testing the hypotheses of others with the goal of joint publication rather than independently developing similar hypotheses, then working in silos towards separate publications. Web-based applications would be further developed for the specific purpose of supporting research cooperation. Telematik-Plattform für Medizinische Forschungsnet (TMF) in Germany, for example, offers an open-access platform for interdisciplinary exchange as well as cross-project and cross-location cooperation in order to identify and address organizational, legal, ethical, and technological problems of modern medical research [19].

This system enhancement will allow investigators to avoid the politics, secrecy, and inefficiencies that characterize the pursuit of publicly funded research in academic institutions. The current system actually inhibits advances by discouraging investigations in areas that appear to be already locked-up with funded but patented research. Those researchers who sought to restrict the use of their materials and methods in the reproduction of their experiments before, during, or after peer review, would be excluded, retrospectively if necessary, from the scientific research publication community. Researchers should be sufficiently confident in the merit and integrity of their work to provide this kind of cooperative transparency for the sake of facilitating scientific progress. We seek to systematically disassemble and revise the perception of laboratories conducting similar research as “competing” laboratories.

The best way to take down the wall between potentially competitive laboratories is to open the data gates [20]. By sharing data, competitors will transform into collaborators. Grants would generally be awarded to laboratories collaborating in the same area, rather than to individual laboratories. Since results would be reported by more than one group, groups would review each other’s data with great scrutiny because fabricated data would tarnish the reputations of all involved parties on publication. Furthermore, with the availability and use of more complete datasets from past studies, future studies could be more efficiently constructed. The “open access” data model would apply to both published as well as unpublished data. Data can remain unpublished for a variety of reasons that include lack of submission or rejection. Researchers may not want to share certain findings or they may not be able to publish important negative or confirmatory work. In either case, it is critical that these data also be accessible. While this access presents additional technical and administrative difficulties, in the current environment of continuously improving (and cheaper) data storage, it should not be insurmountable.

## An Entrenched Culture of Competition

Some may argue that competition, as opposed to cooperation, is the engine that drives scientific discovery. For example, one might maintain that fierce competition accelerated the process of Watson and Crick’s solution of the structure of deoxyribonucleic acid (DNA). However, the competitive process can become counterproductive when secrecy transcends honest collegiality and an “end justifies any means” approach is adopted. Open collaboration with Pauling, Franklin, and Wilkins may well have *shortened* the discovery process. In addition, the credit would have been distributed differently or at least, more widely shared. Watson and Crick feared the possibility of Pauling’s latching onto the solution first far more than they welcomed the potential contribution of Pauling’s genius. Indeed, it was not until Watson and Crick obtained unauthorized and questionably ethical access to the crystallographic work of Rosalind Franklin that they were able to correctly deduce the double helical structure [21].

As biomedical science becomes less of an individualistic pursuit, as substantiated by the large author numbers listed for many publications, the primary driver for scientific knowledge must shift from individual glory to group accomplishment. Outstanding individuals can and will still be recognized and rewarded, but their roles may lie primarily in leading and coordinating groups rather than carrying out entire complex projects on their own. For example, promotion and tenure committees have adjusted to the shift to multi-authorship whereas in a prior era, such publications may have been significantly underweighted due to the small fractional contribution of each author.

## Towards Open Continuous Peer Review

All data from taxpayer-supported funding sources would truly be open and freely available to the public. This would include unpublished or privately published data as well as data from papers published in publicly available journals. This would create an environment where diverse groups could access the relevant database(s) to further investigate and validate (or invalidate) the published findings. Unpublished material may contain keys to interpreting the published results in both positive and negative, reliable and unreliable lights. No research finding would be immune from query and possible challenge. The possibility that an investigator’s published material might even be proven false by a very junior researcher or a layperson would spark the due diligence necessary for accurate data collection and a robust aversion to falsified or fabricated data—as well as to those individuals who engage in such practice. The academic promotion process would adjust to this new culture but, except for the recognized rare lone wolf genius, would actually require investigators to participate in research collaboratives. In addition, the reward system (eg, promotions, awards) would more strongly recognize the publication of important negative results and more negatively weigh the publication of research that proved to be unreliable, required retraction, etc. While it is more difficult and time consuming for evaluative bodies to do so, the content of articles should be carefully considered in detail by such committees. This may not be the case when the process rests on a more superficial count of publications along with the impact factors of the journals in which they are published.

Scholarly journals would need to work together in order to specifically prevent the publication of unreliable research. Post-publication vigilance for reliability on the part of journals (akin to the post-marketing life cycles of drugs) would become a fundamental element of the scholarly publication process. Those journals best at tracking research post publication might see their impact factors rise in a manner proportional to their engagement in the process. Even better, perhaps more valid and useful metrics than impact factors, such as a “collaboration index”, could be developed to better represent journal quality. Post-publication vigilance on the part of journals would become a fundamental element of the scholarly publication process.

## Shouldering the Cost of Reliability

Without the unlikely *deus ex machina* intervention of a Gates or a Buffett to fund a new non-profit organization devoted to disinterested auditing for the sake of improving research reliability, the funding would likely have to derive from a consortium of the interested parties. The culture would need to develop the embedded philosophy that these costs are simply a part of “doing business” in the sense of producing reliably performed and reported research. The possible involved actors include the researchers themselves; the “payers”, or the source of funding, including non-profit, governmental, and industrial agencies; journals; universities for academic researchers; and hospital systems and professional societies for medical research. We propose for a lean and efficient organization, perhaps an independent non-profit entity, funded in an acceptably fair fashion by the involved parties, whose mission would be to provide what increasingly appears to be much required oversight to the research enterprise.

There might also be an unintended educational benefit from the auditing system. These datasets could be used as exercise for graduate or undergraduate students in their respective fields to review and validate, and credit could be awarded to students who participate as a means of displaying their scholarship in the field. Such measures may be necessary as statistical and machine learning methodologies continue to evolve, and a number of data and statistical expert types may be required to validate the findings. Current technology allows for the simple collection and transfer of vast amounts of data, and this portability should be a vehicle for broadening the base that view and validate research for publication, application, and dissemination.

There are examples of new journals that support and promote data sharing. *BMJ Open* was launched in 2011 and was the first medical journal to integrate its submission process with the Dryad digital repository, so that data deposition is part of authors’ submission/workflow [22]. Data Dryad is a curated general-purpose repository that makes the data underlying scientific publications discoverable, freely reusable, and citable [23]. It now has integrated data submission for a growing list of journals. *Scientific Data* is a new open-access, online-only publication for descriptions of scientifically valuable datasets [24]. It is currently calling for submissions and was launched in May 2014. In fact, the traditional journal model may not be the only modality for the public transmission of scientific information: data may be provided freely online as in the Genomes Unzipped project [25].

With open-access data, research will be democratized and no longer confined within traditional academic environments or industry-funded laboratories. This will allow interacting groups of investigators to conduct research with varying methods on the same and/or related topics using the same data. This will mitigate false positives based on biases related to researchers’ hypotheses, reduce unnecessary experimental reproduction, and open research fields to fuller participation.

## Crowdsourcing the Validation of Research Findings

Replication will not remove either bias or residual confounding. For observational studies, hidden variables, including provider bias and local medical culture issues, that confound the true relationship between an exposure and an outcome, tend to differ across heterogeneous settings. If the association between an intervention and an outcome remains strong across countries with very different practices, for example, then the probability that this association is noise rather than signal is reduced (vs an observation in a single locale).

A major challenge with observational studies that is not addressed by the size of the data is the presence of residual confounding: there may be characteristics of the patients or the diseases not captured in clinical databases that may explain why patients who got treatment A got better while those that got treatment B did not. The social determinants of health—the conditions in which people are born, grow, live, work, and age, and most importantly, the individual behaviors in response to these variables—are seldom captured in electronic health records (EHR). The sociological factors are becoming increasingly available as large cities build open data platforms. For example, the NYC Open Data repository [26] contains over 1100 datasets spanning Business, City Government, Education, Environment, Health, Housing and Development, Public Safety, Recreation, Social Services, and Transportation. Behavioral data are likewise increasingly captured digitally through mobile phones, tracking of Internet usage (including social media), global positioning system (GPS) devices and other wireless sensors, purchases, and other financial transactions, etc. The challenge, needless to say, is to map these disparate data sources without significant risk to privacy and security.

For translational and clinical research, the additional research scrutiny would set the stage for the development of useful standard thresholds at which a research finding could be considered valid and reliable, for example, after n number of replications or after a particular statistical requirement is achieved. Within a bigger data framework, the threshold standardization would become progressively more valid due to the statistical confidence afforded by larger datasets.

A key ingredient to making this vision successful is efficient crowdsourcing. Models that may be emulated already exist and are efficiently being used by researchers today. The Multi-parameter Intelligent Monitoring in Intensive Care (MIMIC) database is one such successful initiative [27]. The MIMIC database, developed and maintained by the Laboratory of Computational Physiology at the Massachusetts Institute of Technology (MIT), contains health care metrics of over 60,000 de-identified intensive care unit patients admitted to the Beth Israel Deaconess Medical Center (BIDMC). Information is carefully de-identified to minimize identification risks without excessive deletion of information of clinical value. Natural language processing is used to correctly capture the correct and precise meaning of clinical entries that can be difficult to elucidate based on factors such as physician abbreviations and a plethora of contextual modifiers (eg, “rule out”, “suspect”, “history of”). The success of MIMIC at an admittedly relative small scale gives great hope for our ability to similarly use larger clinical databases for both dynamic and retrospective data mining purposes. This database has already been employed in several publications that have brought together frontline clinicians with data scientists and computer engineers, under the guidance of the authors. The Institutional Review Boards of both MIT and BIDMC have approved the use of MIMIC for research purposes. The authors also recently organized the Critical Data Marathon and Conference held at MIT in January 2014 [28]. The conference’s theme was to address concerns that big data will only augment the problem of unreliable research. Professors Jeffrey Drazen, New England Journal of Medicine editor-in-chief, and John Ioannidis, director of the Meta-Research Innovation Center at Stanford, were the keynote speakers. The second MIMIC data marathon was held concurrently at MIT, in London and in Paris on September 2014 and attracted more than 200 participants.

The PCORnet is a new initiative by the Patient-Centered Outcomes Research Institute [29]. It is a database that will consist of 11 clinical data research networks across the country cataloguing primarily EHR data, with some degree of mapping to 18 patient-powered research networks archiving all types of data collected by patients. If all goes well, by September 2015 PCORnet will be a giant repository of medical information from 26-30 million Americans. In the United Kingdom, the National Institute for Health Research Health Informatics Collaboration was launched in November 2013 [30]. Five National Health Service (NHS) trusts are working together to make NHS clinical data more readily available and accessible to researchers, industry, and the NHS community. The main objectives are to develop, design, and provide common infrastructure, standards, and services that will allow users to perform secondary analysis of EHR data in the fields of viral hepatology, acute coronary syndrome, ovarian cancer, renal transplantation, and critical care. But as we have previously emphasized, the value of these large databases hinges on the researchers’ transparency in their methodology and the creation of a continuous and more effective peer review, leading to improvement in method quality with each iteration of analysis and resulting in more reliability.

Data marathons held around open data (including MIMIC) have attracted both students and postgraduate trainees [31]. Closed research networks would expand and evolve, removing the barriers that have made research activity accessible only to a limited group. In effect, research networks would expand exponentially breaking down the walls that have made much research activity accessible to only a relatively small group of academics.

## Open Big Data Hits and Misses

**Figure 1:**
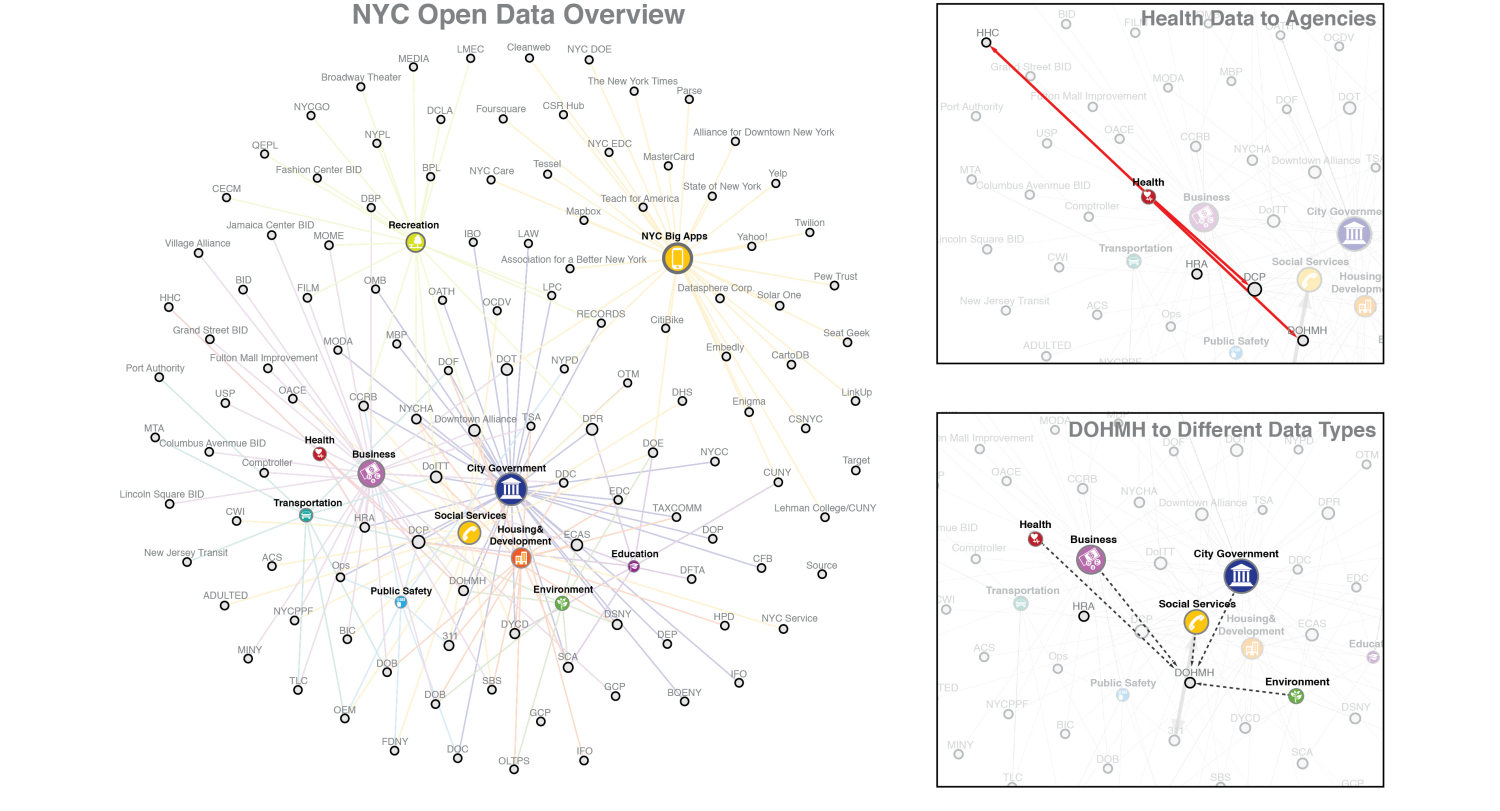
Visualization of NYC Open Data. Figure courtesy of Yuan Lai.

As impressive as it is, the NYC Open Data repository highlights some of the problems that we see in big data initiatives. For example, urban planning has to date not incorporated health care data to fully exploit the data’s potential to inform public health policies. As shown in Figure 1, health data are directly shared only between the Health and Hospitals Corporation, the Department of City Planning, and the Department of Health and Mental Hygiene. Noticeably missing are direct data-sharing connections between these organizations and the New York Department of Sanitation, Department of Homeless Services, Department for the Aging, and the Office of Emergency Management. The association map illustrates that organizations that would benefit from direct multidisciplinary collaboration appear to be operating in informational siloes, which is a recurring theme among big data projects.

But there have been success stories. In October 2012, GlaxoSmithKline announced that it would make detailed data from its clinical trials available to researchers outside its own walls [32]. For a company that spends $6.5 billion a year on research and development, it was a surprising departure from the entrenched system of data secrecy. True to its word, the company began posting its patient-level clinical trial data online in May 2013 and then invited other pharmaceutical companies to do the same. Consequently the Clinical Trial Data Request project was launched [33]. Pharmaceutical companies that have so far committed to contribute (apart from GlaxoSmithKline) include Bayer, Boehringer Ingelheim, Lilly, Novartis, Roche, Sanofi, Takeda, Union Chimique Belge (UCB), and ViiV Healthcare. To date, more than 1000 clinical trials have been uploaded. Trial transparency is appealing because of a growing sense that it could make drug development more efficient, saving the industry billions while also getting breakthrough therapies to patients more quickly.

Finally, the Global Alliance for Genomics and Health was established in 2013 and consists of genomics researchers, funders, businesses, and advocates [34]. The coalition develops and implements technical, ethical, legal, and clinical guidelines to make it easier to share genomic data. This is an example of an international multidisciplinary collaboration around a big data initiative representing various sectors including academia, industry, and the government. The current focus of the group is the creation of a genomics application programming interface to enable the interoperable exchange of data in DNA sequence reads and a framework for data sharing to guide governance and research.

## Implementing Transparent Oversight Systems

**Figure 2:**
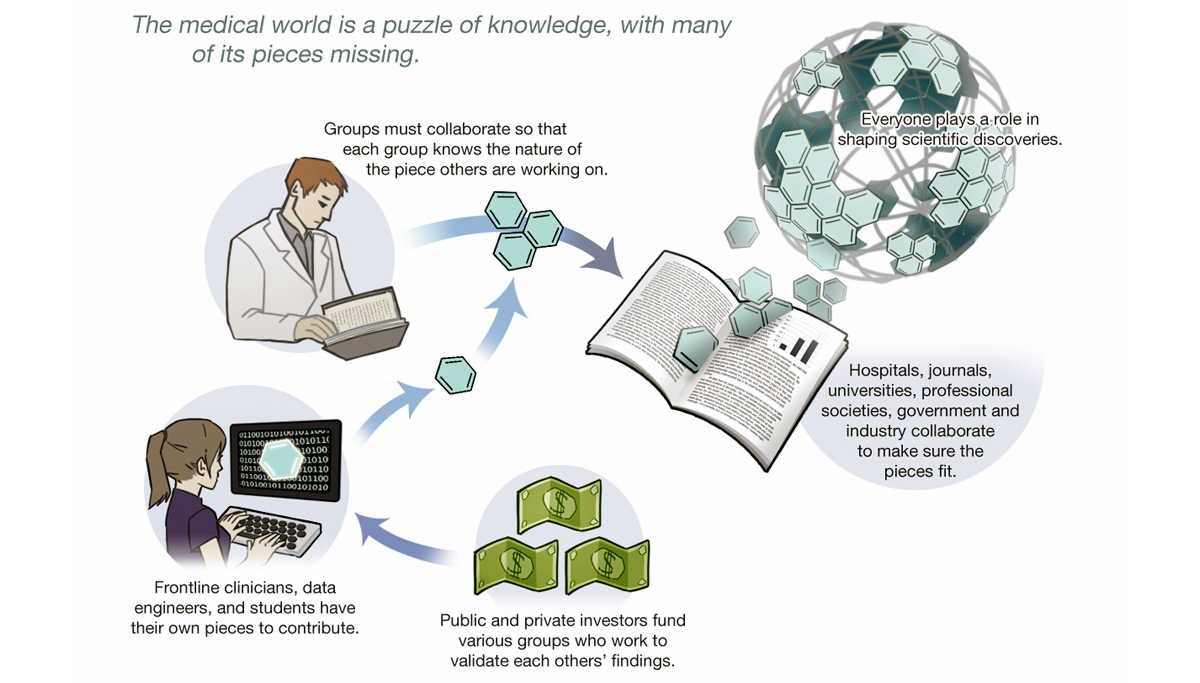
Graphic illustrating how to address unreliable research. Figure courtesy of Kai-ou Tang.

Systems will need to be put in place to institute the replication and cross validation of experiments and analyses (Figure 2). Universities, professional societies, government agencies, and research-driven companies are examples of organizations that could develop and operate these systems. Regulatory boards such as the Food and Drug Administration (FDA) and European Medicines Association (EMA) will have to revise existing approval requirements; for example, trials backing a drug or device will require replication as well as validation by different groups. To renew approval, companies will be required to submit ongoing, regular reports that track the effectiveness of their products in the real world. A pharmacovigilance system has been described that proactively uses clinical database networks to accumulate safety and efficacy evidence when drugs are used in wider, more diverse patient populations than those typically examined during pre-market approval clinical studies [35]. Both the FDA and EMA have already proposed the expansion of data access submitted in regulatory applications [36,37]. In 2012, The Royal Society published a report on science as an open enterprise that mapped out the changes required of scientists, their institutions, and those that fund and support science in order to optimize the potential of the huge deluge of data created by modern technologies [38]. Last year, the AllTrials campaign was launched globally with a cross-sectoral support, calling for all clinical trials to be registered and all results reported [39]. The initiative pushes for researchers, funding bodies, institutions, ethics committees, and regulators to work together to ensure that the value from the resources used to produce research is maximized. In the United States, the Center for Open Science was inaugurated [40]. It is a non-profit technology organization whose mission is to increase the openness, integrity, and of scientific research. At the heart of the organization is the Open Science Framework, an open source software that facilitates collaboration in science research. Most recently, the Meta-Research Innovation Center at Stanford was launched. Headed by Professors John Ioannidis and Steven Goodman, the center will undertake rigorous evaluation of research practices with the aim of optimizing the reliability of scientific investigations and the efficiency of the biomedical research enterprise [41].

With such systems in place, investors will be able to invest in a group of companies working on a related product or idea, rather than solely in individual companies. The ability to invest in a “fund” of related research initiatives should also reduce investor risk in a manner akin to the reduced risk associated with financial index investing versus individual stock picking. Perhaps in the future, new investment products would arise that focus on companies associated with a particular research subject. As noted, funding agencies like the National Institute of Health and the National Science Foundation would award grants to collaborative groups of laboratories. The extensive time and effort spent in preparing grants that may or may not be successfully funded would presumably be reduced, allowing scientists to focus their efforts on research.

The solution to the conundrum of unreliable research lies not only in complete transparency, but more importantly in cooperation among investigators, and with a more lateral distribution of investments, grant funding, and credit for scientific discoveries. We expect these proposals would bring about a culture of collaboration and shared data as well as more complete and accurate reporting of scientific findings. The added accuracy of the scientific findings is only one of the benefits of the systematization of data interrogation. Another will be the enhanced ability of individuals of every educational level and area of expertise to thrust themselves into the fray and contribute to science. We wish to echo the sentiments of Louis Pasteur when he stated, “Science knows no country, because knowledge belongs to humanity, and is the torch which illuminates the world.”

## Abbreviations

BIDMC: Beth Israel Deaconess Medical Center
EHR: electronic health record
EMA: European Medicines Association
FDA: US Food and Drug Administration
MIMIC: Multi-parameter Intelligent Monitoring in Intensive Care
MIT: Massachusetts Institute of Technology
NHS: National Health Service
